# Effects of pitavastatin on walking capacity and CD34^+^/133^+^ cell number in patients with peripheral artery disease

**DOI:** 10.1007/s00380-017-0988-1

**Published:** 2017-05-02

**Authors:** Kenshiro Arao, Takanori Yasu, Yasuhiro Endo, Toshikazu Funazaki, Yoshimi Ota, Kazunori Shimada, Eiichi Tokutake, Naoki Naito, Bonpei Takase, Minoru Wake, Nahoko Ikeda, Yasuto Horie, Hiroyuki Sugimura, Shin-ichi Momomura, Masanobu Kawakami, Investigators of the Anti-Arteriosclerosis and Lipid Lowering with Pitavastatin Evaluation Study in Nippon (ALPEN)

**Affiliations:** 1 First Department of Integrated Medicine, Saitama Medical Center, Jichi Medical University, Saitama, Japan; 2 Department of Cardiovascular Medicine and Nephrology, Dokkyo Medical University Nikko Medical Center, 632 Takatoku Nikko, Tochigi, 321-2593 Japan; 3 Division of Cardiovascular Medicine, Saiseikai Kurihashi Hospital, Saitama, Japan; 4 Division of Cardiovascular Medicine, Saiseikai Kawaguchi General Hospital, Saitama, Japan; 5 Department of Cardiology, Juntendo University School of Medicine, Tokyo, Japan; 6 Division of Cardiology, Tokutake Clinic, Saitama, Japan; 7 Division of Cardiovascular Medicine, National Defense Medical College, Saitama, Japan; 8 Department of Cardiovascular Medicine, Okinawa Chubu Hospital, Okinawa, Japan; 9 Department of Cardiology, Dokkyo Medical University Nikko Medical Center, Tochigi, Japan

**Keywords:** Exercise, Peripheral artery disease, Statins, CD34^+^/133^+^ cells

## Abstract

This multi-center prospective non-randomized comparative study investigated the effects of pitavastatin in patients with peripheral artery disease (PAD) in terms of exercise tolerance capacities and peripheral CD34^+^/133^+^ cell numbers. At baseline, a peripheral blood test was administered to 75 patients with PAD, along with a treadmill exercise test using the Skinner–Gardner protocol to measure asymptomatic walking distance (AWD) and maximum walking distance (MWD). Each patient was assigned to a 6-month pitavastatin treatment group (*n* = 53) or a control group (*n* = 22), according to the patient’s preference. The tests were repeated in both groups at 3 and 6 months. Baseline AWD and MWD correlated positively with the ankle-brachial pressure index (*r* = 0.342, *p* = 0.0032 and *r* = 0.324, *p* = 0.0054, respectively). Both AWD and MWD values improved at 3 and 6 months compared with baseline, and the degrees of their improvement were higher in the pitavastatin treatment group. CD34^+^/133^+^ cell numbers did not change over time or between groups. Eighty-seven percent of patients in the treatment group attained low-density lipoprotein cholesterol levels below 100 mg/dL after 3 months. The study shows that pitavastatin may be effective in increasing exercise tolerance capacity in patients with PAD.

## Introduction

Peripheral artery disease (PAD), the prevalence of which increases with advancing age and reaches approximately 6% in late sixties, occasionally results in poor or even fatal clinical outcomes because of concomitant coronary artery disease (CAD) or cerebrovascular disease [1, 2]. Critical limb ischemia, often leading to lower limb amputation, compromises activities of daily living and lowers overall life prognosis [3, 4]. According to the Trans-Atlantic Inter-Society Consensus for the Management of Peripheral Arterial Disease (TASC II) [5], exercise prior to revascularization is recommended in patients with non-critical limb ischemia PAD. For pharmacotherapy, cilostazol is recommended as Class I agent [5–7]. Statins were previously recommended as Class IIa agents [5, 6]; however, the most recent American Heart Association/American College of Cardiology (AHA/ACC) guideline recommends the use of statins as Class I agents, because statin therapy improves both cardiovascular and limb outcomes in patients with PAD [7]. TASC II and AHA/ACC guidelines recommend that low-density lipoprotein cholesterol (LDL-C) levels in patients with PAD not exceed 100 mg/dL [4, 5, 7]. Statins have been reported to be effective in primary and secondary prevention of coronary arterial events by lowering lipids and exerting various pleiotropic effects, such as improvement of endothelial function and neovascularization [8] following the increase in number and function of endothelial progenitor cells (EPCs) [9, 10], improvement of arterial endothelial function [11–14], a decrease in oxidative stress [14, 15], and regression of plaque volume by non-steroidal isoprenoid products [8, 16–19]. Sata et al. [8] demonstrated that pitavastatin, cerivastatin, and fluvastatin inhibited atherosclerotic lesion progression in apolipoprotein E-deficient mice, while augmenting blood flow recovery and capillary formation in ischemic hind limbs. These results suggest that statins may not promote the development of cancer and atherosclerosis at doses that augment collateral flow growth in ischemic tissues. Furthermore, an increase in EPC numbers by exercise can lead to angiogenesis or neovascularization [20–22]. Despite the TASC-II recommendation for statin therapy in patients with PAD, few studies on using statins in these patients have been reported, and the body of evidence backing this recommendation is not robust [23–25]. Thus, we characterized the effects of pitavastatin on exercise tolerance capacity and the numbers of peripheral CD34^+^/133^+^ progenitor cells in patients with PAD.

## Methods

### Study patients

Inclusion criteria were patients with PAD; age from 45 to 80; more than 3 months after initiation of antiplatelet agents; LDL-C levels: 100–160 mg/dL, and no statin therapy. Patients with PAD were selected from the outpatient clinic chart in each participating institution. All patients demonstrated either ankle-brachial pressure index (ABI) below 0.90 or significant organic stenosis (≥75% diameter) in iliac, femoral, or popliteal arteries using arteriography, computed tomography, Doppler ultrasonography, or magnetic resonance. Exclusion criteria included PAD with Fontaine stage IV; unstable angina; left ventricular ejection fraction <50%; current smoking habit; difficulty participating in the exercise test owing to neurological, ophthalmological, or orthopedic dysfunction; having more than one acute myocardial infarction, cerebrovascular event, peripheral artery revascularization by catheter angioplasty, or bypass grafting within the preceding 3 months; taking LDL apheresis. We recruited 75 patients with PAD.

### Ankle-brachial pressure index (ABI)

The ABI is a rapid, non-invasive, and reliable measurement method that detects and quantifies PAD [26]. The sensitivity of the ABI to detect PAD has been reported in clinical trials to be approximately 95%, with a specificity near 100% [26, 27]. ABI was measured in each subject after a 5-min rest period in the supine position. A cuff was attached to the brachial artery to measure systolic blood pressure. Another cuff was placed around the ankle. The blood pressure cuff was inflated to 20 mmHg above the systolic blood pressure and deflated over the artery in 2-mm/s increments. The ABI was calculated in each leg as the ratio of ipsilateral ankle systolic pressure to the higher of two systolic brachial pressures.

### Testing assay

At baseline and at 3 and 6 months, fasting blood samples were taken from the cubital vein at rest just prior to the treadmill exercise test. We measured levels of total cholesterol (T-Chol), triglycerides (TG), LDL-C, high-density lipoprotein cholesterol (HDL-C), creatine kinase, plasma glucose, glycohemoglobin A1c (HbA1c), plasma insulin, high-sensitivity C-reactive protein (hsCRP), and the number of CD34^+^/133^+^ cells. Parameters of hsCRP and the number of CD34^+^/133^+^ cells were measured at one central location by SRL Co., and all other parameters in each patient’s healthcare institution. T-Chol, TG, HDL-C, LDL-C, and plasma glucose serum levels were measured by enzymatic methods, HbA1c by high-performance liquid chromatography, plasma insulin levels by enzyme immunoassay, and hsCRP by a monoclonal antibody employing latex that allows measurement of samples with low (0.01 mg/dL) to high (42 mg/dL) concentrations (Nanopia CRP; Daiichi Pure Chemicals, Tokyo, Japan). The numbers of CD34^+^/45^+^/133^+^ cells were measured by trained technicians using fluorescence-activated cell sorting (FACS) (FACSCalibur, BD Biosciences, Franklin Lakes, NJ, USA) with anti-human CD34^+^, CD45^+^, and CD133^+^ antibodies (BD Biosciences) as described elsewhere [21, 28]. After gating CD45^+^ cells from whole blood, the numbers of cells double-positive for CD34 and CD133 were measured [21, 28].

### Definition of risk factors

Risk factors were identified from the medical history or hospital data and included HbA1c levels ≥6.5% for diabetes mellitus (DM), T-Chol levels ≥220 mg/dL and/or LDL-C levels ≥140 mg/dL for hypercholesterolemia, systolic blood pressure ≥140 mmHg or diastolic blood pressure ≥90 mmHg for hypertension, and body mass index ≥25 for obesity, as defined by the Japan Society for the Study of Obesity.

### Evaluation of exercise tolerance capacity

At baseline and at 3 and 6 months, the patients underwent a symptom-limited treadmill exercise stress test following the Skinner–Gardner protocol, which consists of a progressive graded workload with a constant speed of 2 miles/h (3.2 km/h) and a 2% increase in grade every 2 min from 0 to 12% [29]. During the treadmill exercise test, standardized verbal encouragement was given, and patients were continuously monitored for hemodynamic response (heart rate, heart rhythm, and blood pressure) to exercise until they experienced leg pain, or until the appearance of angina symptoms. Asymptomatic walking distance (AWD) was calculated as the distance in meters during the treadmill test at the onset of claudication, regardless of whether this symptom manifested as muscle pain, aches, cramps, numbness, or fatigue. Maximum walking distance (MWD) was calculated as the distance walked during the treadmill test before stopping due to claudication.

### Study protocol

The study was a prospective non-randomized comparative multicenter study comparing two PAD groups with or without pitavastatin treatment. Eight healthcare facilities in Japan participated in this study. This protocol was approved by the Ethics Committee of each institution, and written informed consent was obtained from all patients in accordance with the ethical standards of the institutional and the national research committee and with the 1964 Helsinki declaration and its later amendments or comparable ethical standards. Fasting blood and physiological tests were administered between 9:00 AM and 11:00 AM at each outpatient department. At baseline, blood and urine samples were collected from all patients, the ABI was calculated, and the treadmill test was conducted to evaluate each patient’s exercise tolerance capacity, AWD, and MWD [29]. Peripheral blood parameters included blood chemistry, lipid profiles, and CD34^+^/133^+^ cell numbers as a marker for endothelial progenitor cells, the measurement of which has been described elsewhere [21, 28]. After giving general information about PAD to each participant and taking baseline laboratory and physiological measurements, all patients were divided into two groups according to their individual choices: A 6-month pitavastatin treatment group (P, *n* = 53), and a control group that did not receive any statins (C, *n* = 22). The daily dose of pitavastatin was prescribed by each patient’s physician according to the TASC-II guideline (LDL-C below 100 mg/dL) [5]. Following group assignment, the tests were repeated in both groups at the 3- and 6-month marks. During the study period, all patients were advised to maintain a consistent frequency and intensity of daily exercise as far as possible, and to record their daily walking time and pedometer counts in an exercise journal [30].

### Statistical analysis

Data were analyzed using JMP 7.01J software (SAS Institute, Cary, NC, USA). Continuous variables are expressed as mean ± standard deviation (SD). Mean values were compared between two groups, where appropriate, by Student’s *t* test or two-way analysis of variance followed by the Tukey–Kramer post hoc test. Categorical variables are expressed as frequencies and were compared using Chi-squared analysis. Correlations were assessed using Fisher’s coefficient (*r*). Probability values of *p* < 0.05 were considered statistically significant.

## Results

During the study period, three patients withdrew because of major adverse events in the control group: Subarachnoid hemorrhage (*n* = 1), brain stem cerebral embolism (*n* = 1), and intestinal bleeding due to rectal cancer (*n* = 1). The other 72 patients completed the study protocol without any cardiovascular events. In the statin treatment group, no adverse events occurred during the study. In the control group, medication regimens were not altered during the study period. Baseline patient characteristics are shown in Table 1. T-Chol, HDL-C, and LDL-C levels were similar between the two groups, but HbA1c and TG levels, as well as percentiles of ischemic heart disease and diabetes, were higher in the statin group than in the control group.

**Table 1:** Clinical characteristics of study patients

	Pitavastatin (*n* = 53)	Control (*n* = 22)	*p*
Age (years)	70 ± 7	71 ± 7	0.859
Gender (M/F)	39/14	19/3	0.366
Lower ABI	0.73 ± 0.15	0.74 ± 0.20	0.811
Body mass index	23.0 ± 3.1	22.7 ± 2.8	0.671
Hemodialysis (±)	5/48	2/20	0.963
Hypertension (±)	36/17	11/11	0.223
Diabetes (±)	36/17	4/18	0.0002*
Ischemic heart disease (±)	30/23	5/17	0.0015*
Smoking status (±)	14/39	4/18	0.366
Glycohemoglobin A1c (%)	6.3 ± 1.1	5.6 ± 0.5	0.0048*
Total cholesterol (mg/dL)	207 ± 40	193 ± 39	0.181
Triglycerides (mg/dL)	154 ± 82	115 ± 51	0.046*
HDL-C (mg/dL)	51 ± 14	52 ± 12	0.770
LDL-C (mg/dL)	123 ± 32	115 ± 38	0.325
hsCRP (mg/dL)	0.31 ± 0.60	0.22 ± 0.23	0.593
Log_10_CD34^+^/133^+^ cells (100 μL)	1.67 ± 0.29	1.73 ± 0.29	0.259
Treadmill exercise test			
Asymptomatic walking distance (m)	233 ± 207	211 ± 166	0.5853
Maximum walking distance (m)	473 ± 345	433 ± 321	0.5205
Daily exercise (steps/day)	5757 ± 2524	6183 ± 3680	0.8146

Values are expressed as mean ± SD or number of participants in that category. Statistical significance was defined as * *p* < 0.05

*ABI* ankle-brachial pressure index, *HDL-C* high-density lipoprotein cholesterol, *hsCRP* high-sensitivity C-reactive protein, *LDL-C* low-density lipoprotein cholesterol

**Fig. 1:**
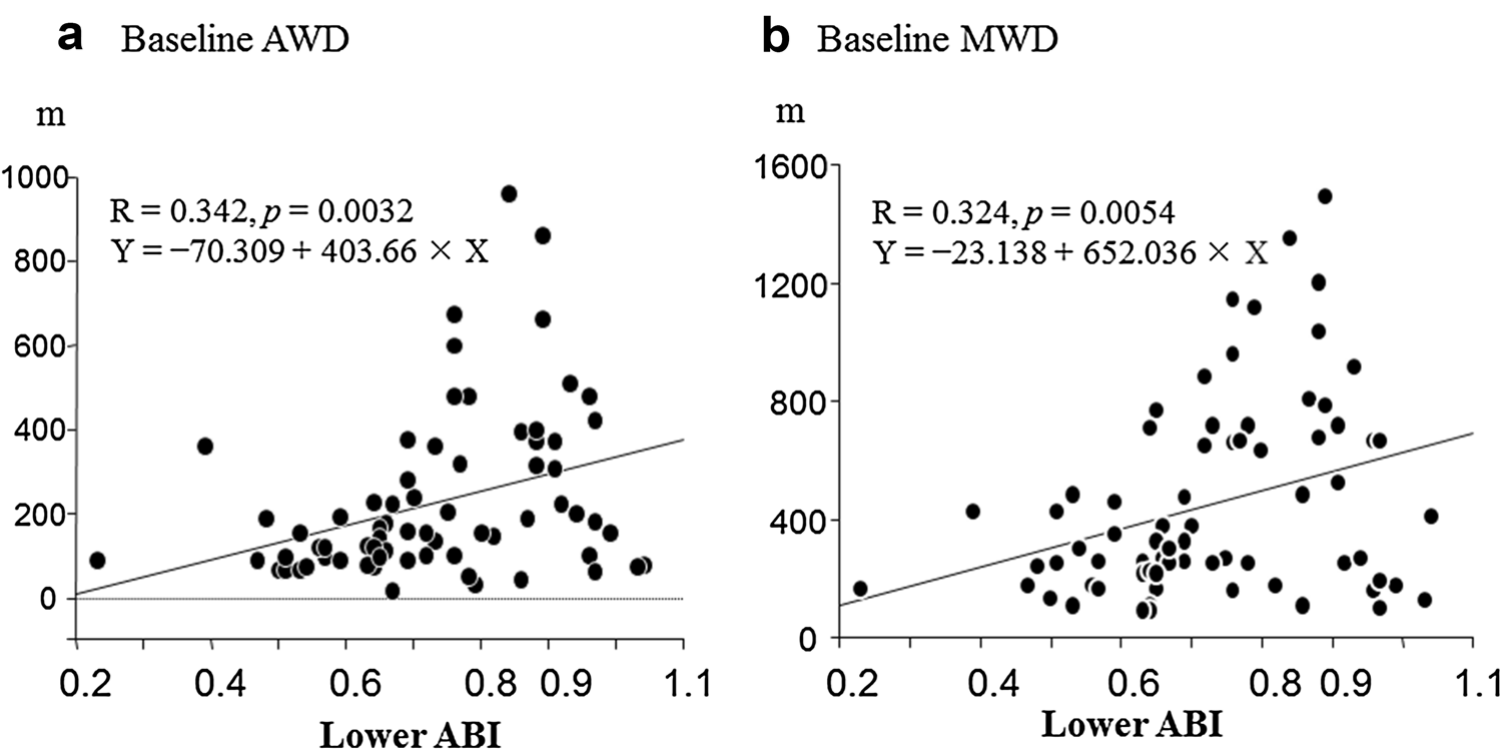
Baseline correlations of ankle-brachial pressure index (ABI) with exercise tolerance parameters in study patients. *AWD* asymptomatic walking distance, *m* meters, *MWD* maximum walking distance

**Fig. 2:**
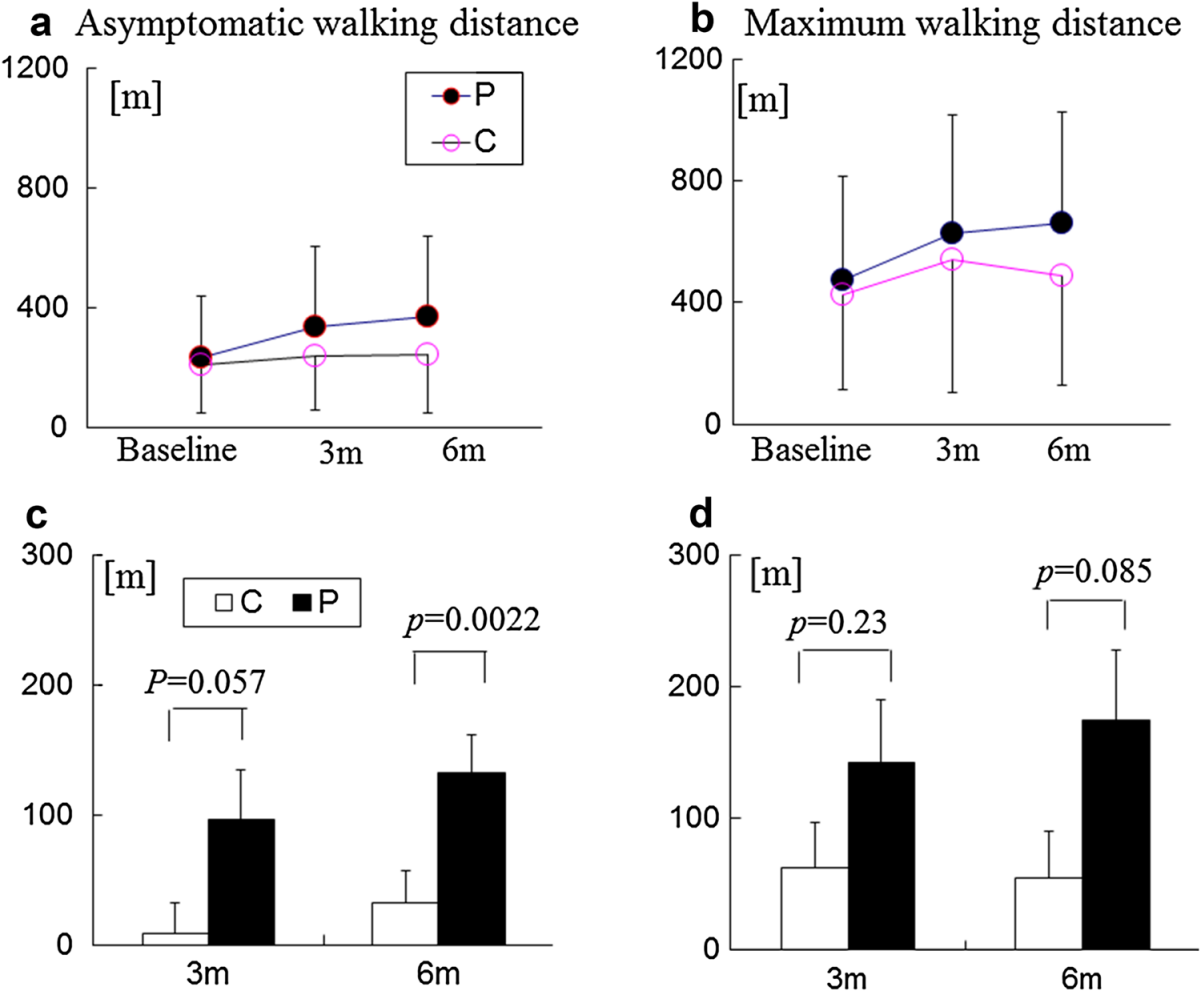
Changes in exercise tolerance capacity in the treadmill exercise test at 3 and 6 months. Serial changes in asymptomatic walking distance (**a**) and maximum walking distance (**b**), and absolute changes in asymptomatic walking distance (**c**) and maximum walking distance (**d**) from baseline. *3* *m* 3 months, *6* *m* 6 months, *C* control group, *m* meters, *P* pitavastatin treatment group

**Fig. 3:**
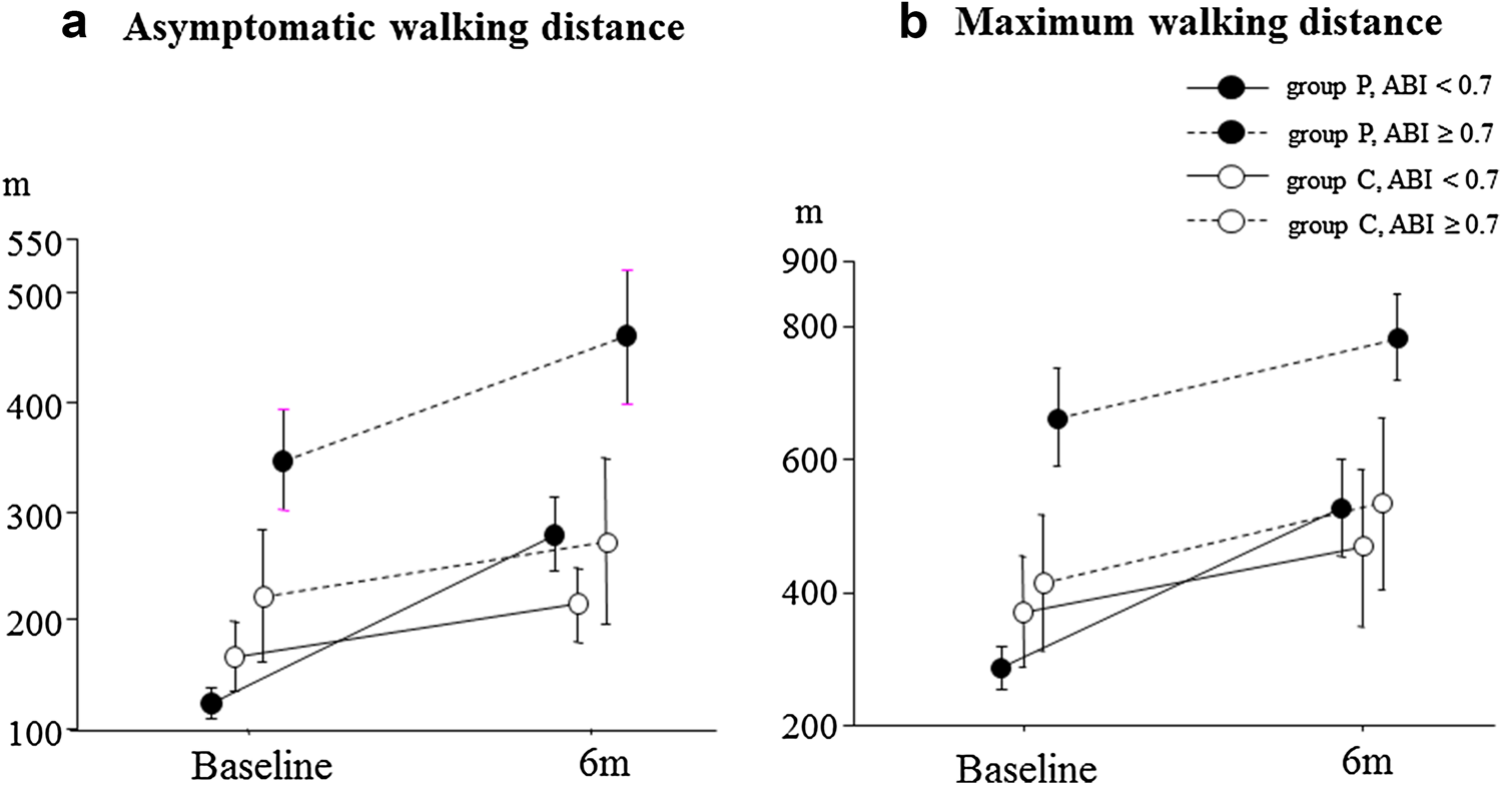
Serial changes in exercise tolerance capacity parameters between baseline and the 6-month mark. Absolute changes in asymptomatic walking distance (AWD) and maximum walking distance (MWD) compared with baseline at 6 months. *ABI* ankle-brachial pressure index, *C* control group, *P* pitavastatin treatment group

Table 2 displays the serial changes in blood parameters. AWD and MWD values for the baseline treadmill exercise test correlated with ABI (AWD: *r* = 0.342, *p* = 0.0032; MWD: *r* = 0.324, *p* = 0.0054; Fig. 1). Daily doses of pitavastatin ranged as follows: Four milligrams (6% of patients), 2 mg (60% of patients), 1 mg (30% of patients), and 0.5 mg (4% of patients). At the 6-month mark, lipid parameters improved in the statin group, and 87% of patients in the group achieved the recommended TASC-II guideline (LDL-C below 100 mg/dL). At 3 and 6 months, both AWD and MWD in the statin group improved compared with the group’s baseline values (AWD: *p* = 0.0013; MWD: *p* = 0.0004). AWD and MWD values in the control group did not change significantly over time (Fig. 2). At 6 months, both AWD and absolute change in AWD were greater in the statin group than in the control group, and MWD in the statin group was generally greater than in the control group (Fig. 3).

**Table 2:** Serial changes in lipid parameters, hsCRP, and CD34^+^/133^+^ cell numbers

	Pitavastatin (*n* = 53)				Control (*n* = 22)			
	Baseline	3 M	6 M	*p* value	Baseline	3 M	6 M	*p* value
Lipid parameters								
T-Chol (mg/dL)	207 ± 40	158 ± 29	159 ± 29	<0.0001	193 ± 39	197 ± 39	196 ± 43	0.215
TG (mg/dL)	154 ± 82	124 ± 81	126 ± 88	0.005	115 ± 51	101 ± 45	103 ± 42	0.057
HDL-C (mg/dL)	51 ± 14	53 ± 13	53 ± 12	0.220	52 ± 12	53 ± 14	55 ± 15	0.065
LDL-C (mg/dL)	123 ± 32	79 ± 22	82 ± 21	<0.0001	115 ± 38	131 ± 37	116 ± 42	0.274
hsCRP (mg/dL)	0.31 ± 0.60	0.38 ± 0.70	0.17 ± 0.15	0.056	0.22 ± 0.23	0.23 ± 0.23	0.23 ± 0.26	0.577
Log_10_CD34^+^/133^+^ cells	1.67 ± 0.29	1.73 ± 0.31	1.74 ± 0.29	0.923	1.73 ± 0.29	1.65 ± 0.24	1.68 ± 0.31	0.259

Values are expressed as mean ± SD. Statistical significance was defined as * *p* < 0.05

*HDL-C* high-density lipoprotein cholesterol, *hsCRP* high-sensitivity C-reactive protein, *LDL-C* low-density lipoprotein cholesterol, *M* months, *T-Chol* total cholesterol, *TG* triglycerides

**Fig. 4:**
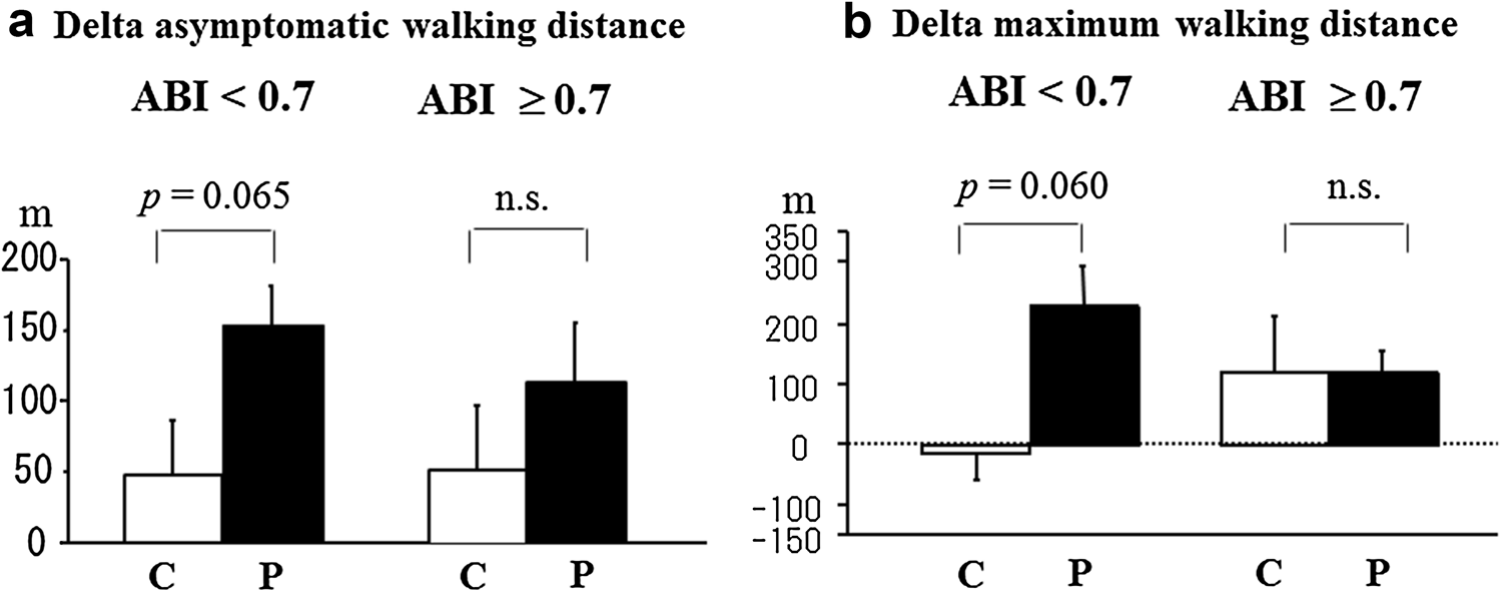
Absolute changes in exercise tolerance parameters following a 6-month treatment. *ABI* ankle-brachial pressure index, *C* control group, *n.s.* not significant, *P* pitavastatin treatment group

**Fig. 5:**
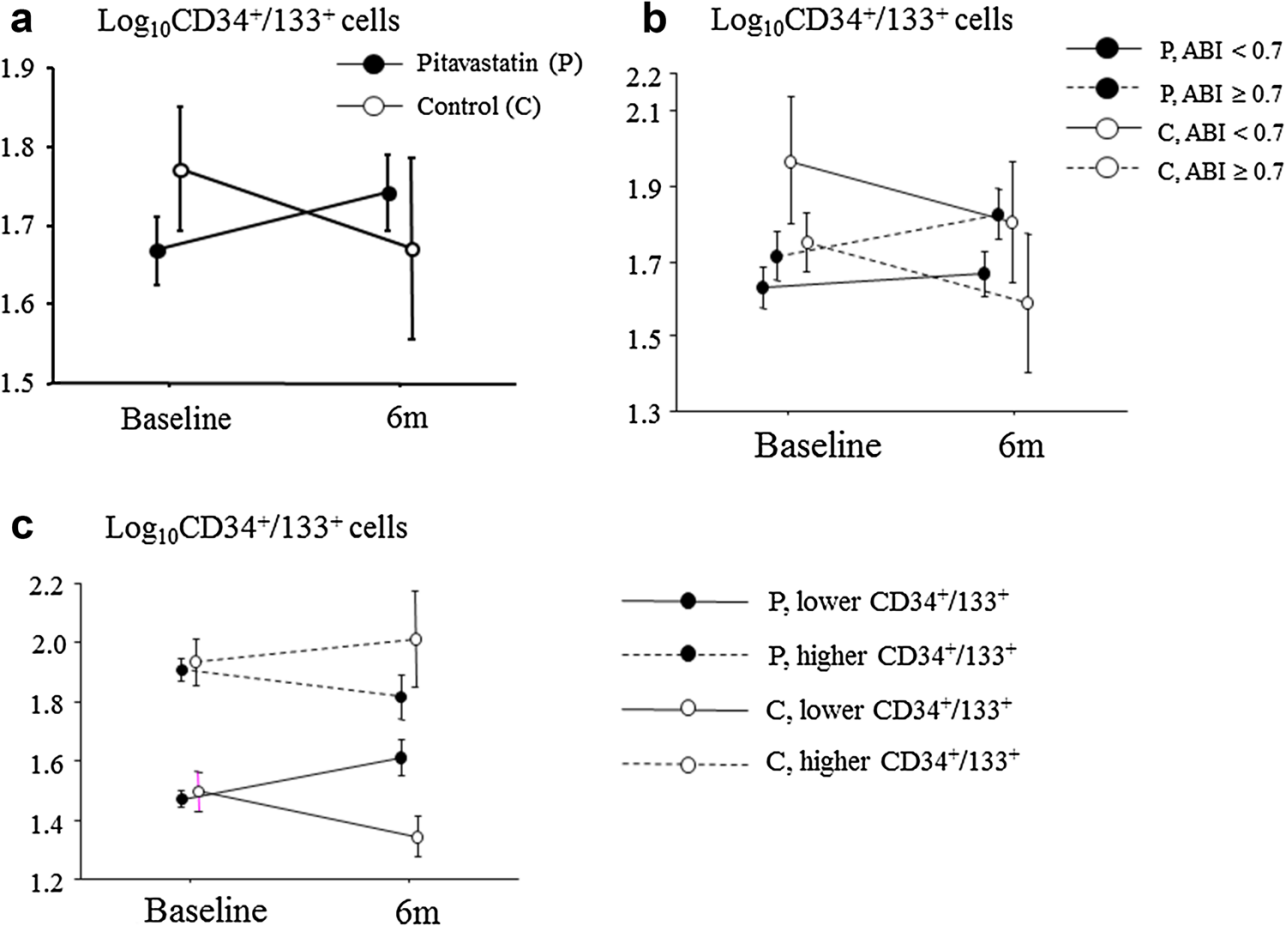
Serial changes in peripheral Log_10_CD34^+^/133^+^ cell numbers following a 6-month treatment. **a** Comparison of the pitavastatin treatment group (*P*) and the control group (*C*). **b** Comparison between groups by treatment and ankle-brachial pressure index (ABI). **c** Comparison between groups by treatment and Log_10_CD34^+^/133^+^ cell numbers

Furthermore, in patients with ABI below 0.7, the degrees of improvement in exercise tolerance capacity in the statin group were significantly greater than those in the control group (Fig. 4). hsCRP as a marker for inflammation decreased after pitavastatin treatment, especially in patients with diabetes. The number of CD34^+^/133^+^ cells did not change significantly in either group, and the two groups did not differ (Table 2; Fig. 5).

## Discussion

In the present study, we defined exercise capacity by the AWD and MWD at baseline, and found that both values were greater in the statin group after 6 months, and were accompanied by effective lipid lowering. The numbers of CD34^+^/133^+^ cells did not change significantly over time and did not differ between groups.

### Baseline correlation of ABI with exercise tolerance capacity

In the baseline treadmill test, exercise tolerance capacities, measured as AWD and MWD, were positively correlated with lower baseline ABI values. This finding may be leveraged to estimate the approximate length and intensity of daily exercise as therapy.

### Changes in exercise tolerance capacities after a 6-month pitavastatin treatment

In the pitavastatin treatment group, the two exercise capacity parameters, AWD and MWD, improved over time, whereas those in the control group did not change significantly. The absolute change in AWD at 6 months was greater in the statin group than in control the control group. Exercise improves walking performance in patients with PAD [4, 5, 30]. The effect of statins on walking performance in patients with PAD varies in published studies, but most researchers concurred that statins improve walking performance [23, 24]. Our findings concur with those reported by Mohler et al., who used atorvastatin to improve walking distance in patients with PAD [24].

Furthermore, in patients with ABI below 0.7 (32 of 75 patients), the absolute changes in AWD and MWD in the statin group were significantly greater than those in the control group. Although the sample size was small, this finding may suggest the greater efficacy of statins in patients with PAD with moderate to severe arterial stenosis than in those with only mild stenosis. The present study indicates that statins may be more effective in patients with low ABI values in terms of exercise tolerance capacity. Statins exert pleiotropic effects such as plaque stability, plaque volume regression, and anti-inflammatory action [8, 16–19, 31]. One possible explanation is the plaque volume regression at the culprit lesion. In the pitavastatin group, ABI values below 0.7 increased after a 6-month statin treatment (*p* = 0.0004, data not shown), while no significant change was observed in patients with ABI over 0.7 regardless of changes in LDL-C, HDL-C, and hsCRP. Lesions with lower ABI exert higher plaque burdens, and plaque regression and stability by statin treatment may lead to improvement of ABI and exercise tolerance capacity.

### CD34^+^/133^+^ cell numbers in peripheral blood following statin treatment

In contrast with our expectations, the number of CD34^+^/133^+^ cells did not increase significantly after a 6-month statin treatment. According to previous reports, exercise [9, 10, 21, 32] or statin treatment [23, 24] can increase the numbers of peripheral EPCs, while diabetes can decrease them [33]. Exercise increases the number of EPCs because increased vascular shear stress boosts the production of nitric oxide [9, 10, 21]. Statin treatment can induce the movement of EPCs from bone marrow to peripheral blood [23, 24] and augment collateral flow growth in ischemic tissues [8]. It is possible that our sample size was too small to detect differences. In addition, patients presented with individual effect modifiers, such as advancing age, DM, and varying levels of daily activity; all these factors may have influenced the number of CD34^+^/133^+^ cells. A randomized study with a much larger sample size would therefore be required.

### Risk factors and PAD

As shown in Table 1, most patients had coronary risk factors, and more than half (40 of 75) of these patients with PAD also had CAD, a percentage similar to that in previous reports [5, 23, 24]. Statins have been reported to assist in lipid management in patients with CAD and PAD [16–19, 31, 34]. Though each patient in our study made an individual decision regarding statin treatment after receiving a general explanation on lipid management, our numbers may reflect the baseline differences in TGs and percentage of concomitant CAD between the two groups. Thus, the patient background could not be equalized between the statin and control groups.

### Achievement of LDL-C targets after a 6-month pitavastatin treatment

Eighty-five percent of patients with PAD treated with pitavastatin for 6 months achieved the recommended serum LDL-C target (under 100 mg/dL) without any significant side effects [5]. Overall adherence to statin administration was high in all groups (>95%). This pilot study suggests that oral administration of pitavastatin for 6 months is feasible in terms of safety and tolerance. Additionally, the findings indicate that pitavastatin may offer a modest benefit for functional capacity, as determined by initial and absolute claudication distance and self-reported walking speed. Ninety percent of patients with PAD in the statin group took a 1- or 2-mg daily dose of pitavastatin, and the maximal dose was 4 mg; these doses are standard in Japanese patients with atherosclerosis. Furthermore, CAD comorbidity is typically found in approximately 50% of patients with PAD, and is also treated by controlling lipid levels [5]. Use of a strong statin may reduce the need for revascularization, but reductions in rates of amputation were not observed in the previous studies [25]. Moderate-dose statin therapy is safe, and the minor risks are greatly outweighed by the benefits.

### Other improvements in blood parameters following pitavastatin treatment

We found that pitavastatin treatment showed a tendency toward a decrease in inflammation, as measured by levels of hsCRP, especially in patients with diabetes; this was not observed in the control group. The present results regarding hsCRP reduction by strong statins are in line with the previous reports [35–37].

## Conclusions

This pilot study, although small, showed that a 6-month treatment with pitavastatin is feasible in terms of safety and efficacy in managing serum lipid levels and improving exercise tolerance capacity in patients with PAD.

### Study limitations

This study was conducted as a pilot study prior to a randomized, double-blind cohort study. Baseline patient characteristics varied, because patients were assigned not randomly but according to their own preferences, leading to a possible selection bias. To confirm our findings, a larger patient cohort assigned randomly to treatment groups would be required. In addition, patients engaged in non-supervised daily exercise, and the intensity and duration of exercise varied by patient. Third, maximal exercise distance by the treadmill test was determined objectively rather than subjectively, because the test was terminated at the discretion of the patient. Finally, we evaluated EPCs in peripheral blood as the numbers of CD34^+^/133^+^ cells, although other indices for EPC can be used in clinical settings [22, 38].
